# Acute ischemic heart disease and interventional cardiology: a time for pause

**DOI:** 10.1186/1741-7015-4-25

**Published:** 2006-10-11

**Authors:** Peter Bogaty, James M Brophy

**Affiliations:** 1 Quebec Heart Institute/Laval Hospital, Laval University, 2725 Chemin Ste-Foy, Quebec, G1V 4G5, Canada; 2 McGill University Health Center, McGill University, Montreal, Canada

## Abstract

**Background:**

A major change has occurred in the last few years in the therapeutic approach to patients presenting with all forms of acute coronary syndromes. Whether or not these patients present initially to tertiary cardiac care centers, they are now routinely referred for early coronary angiography and increasingly undergo percutaneous revascularization. This practice is driven primarily by the angiographic image and technical feasibility. Concomitantly, there has been a decline in expectant or ischemia-guided medical management based on specific clinical presentation, response to initial treatment, and results of noninvasive stratification. This 'tertiarization' of acute coronary care has been fuelled by the increasing sophistication of the cardiac armamentarium, the peer-reviewed publication of clinical studies purporting to show the superiority of invasive cardiac interventions, and predominantly supporting (non-peer-reviewed) editorials, newsletters, and opinion pieces.

**Discussion:**

This review presents another perspective, based on a critical reexamination of the evidence. The topics addressed are: reperfusion treatment of ST-elevation myocardial infarction; the indications for invasive intervention following thrombolysis; the role of invasive management in non-ST-elevation myocardial infarction and unstable angina; and cost-effectiveness and real world considerations. A few cases encountered in recent practice in community and tertiary hospitals are presented for illustrative purposes The numerous and far-reaching scientific, economic, and philosophical implications that are a consequence of this marked change in clinical practice as well as healthcare, decisional and conflict of interest issues are explored.

**Summary:**

The weight of evidence does not support the contemporary unfocused broad use of invasive interventional procedures across the spectrum of acute coronary clinical presentations. Excessive and unselective recourse to these procedures has deleterious implications for the organization of cardiac health care and undesirable economic, scientific and intellectual consequences. It is suggested that there is need for a new equilibrium based on more refined clinical risk stratification in the treatment of patients who present with acute coronary syndromes.

## Background

Over the past few years, a major change has occurred in the approach to patients presenting with acute coronary syndromes (ACS), from predominantly expectant medical management, based on specific clinical presentation, response to initial treatment, evolution, and results of noninvasive risk stratification, towards rapid and increasingly systematic coronary angiography. This change has paved the way to percutaneous coronary revascularization (PCI), largely driven by angiographically perceived coronary anatomy and technical feasibility. Clinical success tends to be measured in terms of the physician's satisfaction with the immediate angiographic result of the intervention, ever easier and safer to assure because of the improving skills of interventional cardiologists and the increasing sophistication of the cardiac armamentarium. Tertiary hospitals and interventional cardiologists are understandably eager to extend these services to community and regional hospitals, since, beyond the conviction that this is the best approach, this also reinforces their *raison d'être*, justifying claims for more new high-tech resources and, depending on the socioeconomic environment, potentially increasing revenues. Trainees in tertiary academic centers are becoming conditioned to the 'culture of intervention' as the natural way to practice cardiology. Clinicians in non-tertiary centers increasingly refer their patients for invasive management so as not to deprive them of this new standard of care. While progress in the field of interventional cardiology has undoubtedly had a beneficial impact on the ability to manage acute coronary disease, the skeptic may be struck by how an invasive cardiac approach has become the norm, predicated less on clear clinical need and increasingly on the presumption that this is the best thing to do, the technological imperative. In the design and interpretation of clinical trials, and in reviews, opinion pieces, and editorials, the contemporary medical literature has predominantly supported, indeed reinforced, this tendency [1-5]. We believe there is need for another perspective. This article, using typical cases culled from our routine practice, examines the evidence on which the current invasive approach to ACS is based, and explores its far-reaching clinical, economic, and intellectual implications.

## Discussion

### Choice of initial reperfusion therapy in ST-elevation myocardial infarction (STEMI)

#### Case 1

*A 75-year-old woman presented at a regional hospital with a 2-hour history of increasing chest discomfort. The electrocardiogram *(*ECG*)*showed 3-mm ST-segment elevation in inferior leads. The patient was given aspirin and heparin and transferred 30 miles by ambulance to a tertiary center for primary angioplasty. Coronary angiography was begun 2.5 hours later and showed single-vessel disease, a 70% right coronary artery ostial stenosis with TIMI-3 flow. Angioplasty was performed with insertion of three stents with an intravenous infusion of the platelet glycoprotein IIb/IIIa blocker, abciximab. The patient was re-transferred the next day and recovered uneventfully*.

This case raises the issues of unseemly treatment delays on outcomes and the rationale for intervention when apparent reperfusion (TIMI-3 flow) has occurred.

The widely accepted notion that primary angioplasty (PA) is superior to thrombolysis is based on the recent meta-analysis of 23 randomized trials by Keeley *et al *showing a 2% mortality advantage for angioplasty (7% versus 9%, *p *= 0.0002) [6]. However, the overall conclusion of this meta-analysis may be questioned as older trials, using less efficient thrombolytic strategies, and one large trial (SHOCK) not comparing thrombolysis to PA were included. Thus, when the 11 trials using rapidly administered, but not bolus, fibrin-specific agents are considered separately, a significant advantage for angioplasty is either modestly present (*p *= 0.018) [6] or no longer so (1.1 lives saved/100 patients treated, 95% CI [minus]0.3/100 to 2.6/100) [7], depending on the analytical methods. When statistical significance hinges on model assumptions, it suggests that evidence of superiority for any reperfusion approach is fragile.

Time is a critical determinant of survival with any reperfusion strategy [8,9]. Each hour of delay to thrombolysis is associated with a loss of at least 1.6 lives/100 treated, and possibly many more if the 'golden' first hour after symptom onset is missed [10,11]. Concordant with these observations, prehospital thrombolysis with a 58-minute reduction in treatment time has been shown to confer a 1.7% absolute reduction in mortality compared with in-hospital thrombolysis [8]. A meta regression analysis from the 23 thrombolysis/PA trials has shown loss of the survival benefit for angioplasty when 'door-to-balloon' time exceeds 'door-to-needle' time for thrombolysis by 60 minutes [12]. Very recent large registry data confirm significant step-ups in mortality for each 30-minute increase in door-to-balloon time beyond 90 minutes [13]. Thus, a coherent body of knowledge underpins the importance of time until treatment and its corollary that in certain situations thrombolysis may be just as effective, than PA, or even more so. This corollary has been further underscored by a recent trial demonstrating improved clinical outcomes with prehospital thrombolysis and judicious recourse to so-called rescue angioplasty compared with PA [14,15].

Questions remain as to the practicality or applicability of published results favorable to PA. The mean delay to balloon inflation in the clinical trials was only 40 minutes but in the real world, delays are far longer and it is not established that similar clinical results may be attained [16-18]. Also, the mortality in the thrombolytic arms of these studies was higher (8–14%) than that noted in contemporary registry and trial data of thrombolytic therapy (5–6%) [17,19,20]. Outcomes with PA depend on volume of activity [16,21] and whether the procedure is performed during or off normal working hours [22], limitations that have not been shown to apply to thrombolysis [16]. While recently updated STEMI guidelines [23] acknowledge that no single reperfusion strategy is appropriate for all patients and emphasize that the timely use of some reperfusion therapy is likely more important than the choice of therapy, these judicious considerations are not reflected in the current climate of enthusiasm with interventional cardiology.

The other two outcomes regarding which PA is claimed to be superior are strokes and myocardial reinfarctions. While there are about three more incapacitating hemorrhagic strokes per 1000 in patients treated with thrombolysis than in those treated with PA, there are about 20 excess major bleeds with angioplasty [6,24]. Bleeding can be more than an inconvenience; it has very recently been found to be independently associated with greater mortality in patients with ACS, both at 30 days and at 6 months. [25] Quantification of reinfarction benefit is confounded by ascertainment bias, differing cardiac enzyme criteria depending on whether reinfarction is spontaneous or PCI-related, unbalanced use of a second antiplatelet agent (generally clopidogrel) that reduces reinfarction [26,27], and the problematic consideration of ST-segment re-elevation in the immediate hours following the initiation of thrombolytic therapy. Although these latter events tend to be counted as reinfarctions, the complications that are mostly unique to primary PCI (no reflow, dissection, distal embolization, and side-branch occlusion) [28] and that might be considered iatrogenic reinfarctions are not systematically recorded in the trials. These limitations underline the uncertainty regarding the pertinence of the reinfarction end point in these comparative trials.

Thus, while a small benefit of PA over optimal fibrin-specific thrombolysis is suggested, uncertainty still clouds this issue and both options remain acceptable. The addition of clopidogrel to thrombolysis is likely to improve the performance of the latter relative to PA [26,27]. A recent regression analysis based on the randomized clinical trials suggests no benefit of PA over thrombolysis when the mortality risk of STEMI is < 4.5% and possible harm with treatment delay [29]. Therefore, discriminating clinicians might reserve the recourse to PA for patients with acute myocardial infarction (MI) at particular risk (patients with heart failure or hemodynamic instability or high bleeding risk from thrombolysis) [17,30,31]. The importance of avoiding excessive delays with hospital transfers and off-hours angioplasty that may compromise patient outcomes compared with prompt on-site thrombolysis cannot be overemphasized. Is it not paradoxical that PA is so highly promoted when it is in fact delivered within the time frames recommended by evidence-based guidelines in only a minority of cases [18]?

### Plasticity of indications for PCI after thrombolysis

#### Case 2

*Twenty-four hours after thrombolysis of a Q-wave inferior MI, a 71-year old man had 5 minutes of mild chest discomfort followed by spontaneous resolution. There were no associated hemodynamic or dynamic electrocardiographic abnormalities noted. An angiogram was ordered and performed the next day. The patient had single-vessel disease with an occluded right coronary artery that was successfully reopened with 2 stents inserted under an infusion of eptifibatide, a platelet glycoprotein IIb/IIIa receptor blocker. There was normal TIMI-3 flow. The contrast ventriculogram showed inferior akinesia with an ejection fraction over 50%. The patient did well subsequently. An echocardiogram a year later showed unchanged inferior akinesia*.

This case raises the issues of whether invasive intervention is indicated for recurrent mild, self-limited symptoms following MI and whether delayed opening of an occluded coronary artery after MI is beneficial, relative to the expenses and risks, given our current understanding of myocardial viability.

Even when the initial approach in STEMI is thrombolysis, the threshold for invasive intervention is becoming ever lower, more as a function of the improved safety of the procedure than proof of improved outcomes. Another common example is the patient sent for 'rescue' angioplasty after thrombolysis because of residual chest pain, despite ST-segment resolution. Persistent but diminishing chest discomfort is common following presentation with acute MI and we believe that it tends not to be distinguished from new or ongoing ischemia. Nor is it appreciated how symptoms may be amplified by the attitude and behavior of the treating team. The 'rescue' coronary angiogram will undoubtedly reveal a culprit lesion that will be stented even if flow is already satisfactory on angiographic imaging, and frequently even if the stenosis is no longer significant. The threshold for this intervention can be expected to fall further as fears of restenosis diminish with drug-eluting stents. The satisfaction with the result–often displayed to the patient in the catheterization laboratory by video rerun–will have a powerful therapeutic effect on residual symptoms. The still photographs often included in the patient chart will serve as a stimulus to clinicians for further rapid referrals.

Another not uncommon example of 'rescue' angioplasty is the asymptomatic or minimally symptomatic patient referred for PCI because of persistent ST-elevation of variable degree, although the time window for any reasonable expectation of myocardial salvage is well past. Poor microvascular perfusion may well persist despite successful stenting, platelet glycoprotein IIb/IIIa blockade, and even TIMI-3 flow [32], suggesting that 'no-reflow' was the problem in the first place and was unchanged by the intervention. Inadequate myocardial perfusion despite reestablished epicardial artery flow, a phenomenon that is ischemic-time-dependent (26), in addition to a low potential for myocardial salvage after a few hours of persistent ischemia, may account for absence of consistent and marked benefit of 'rescue' angioplasty in recent studies [33-35].

In our experience, another group frequently referred for cardiac catheterization and PCI is post-MI patients who perform a very adequate exercise test, indicative of low risk, without ischemic symptoms, but whose ECG shows ST-depression, compatible with residual myocardial ischemia. However, several large studies, notably in the GISSI thrombolytic cohort, have shown that the sole presence of an asymptomatic ECG-positive exercise test following MI predicts neither mortality nor reinfarction [36,37]. Even the more recent DANAMI-1 study [38], with its inherent biases in favor of aggressive intervention (suboptimal medical therapy and inappropriate randomization of high-risk patients), showed only relatively small benefit from intervention in this context [39].

The final example of overuse of invasive intervention in STEMI is the routine recourse to angiography following thrombolysis, resulting in anatomy-driven revascularization of significant and, increasingly, of borderline and noncritical lesions [40]. The belief that coronary angiography is the most secure and definitive means of risk stratification and that most of these patients will need angiography in any case, is being espoused by growing numbers of clinicians, contributing to the surge of PCI following an ACS. This strong faith in and increasing clinical dependence on 'defining the coronary anatomy' is paradoxical, given evidence that 1) coronary anatomy is an unreliable indicator and predictor of symptoms, the functional significance of lesions, and the risk of new or recurrent acute coronary instability; 2) left ventricular function is much more prognostically important than coronary anatomy; and 3) the prognostic capability of noninvasive physiological stratification using the ECG exercise test or stress nuclear perfusion imaging or echocardiography is well established.

Older studies showing that there was no advantage to systematic invasive cardiac management after MI are considered no longer relevant in the modern era [41]. The recent GRACIA study randomized 500 patients with acute STEMI and concluded that a systematic invasive approach is better than an ischemia-guided approach [42]. The primary combined end point of death, reinfarction, and revascularization at 12 months occurred in 21% of the group whose treatment was ischemia-guided, versus 9% of the group whose treatment was invasive (relative risk 0.44, 95% CI 0.28–0.70) and was essentially driven by the revascularization rate. However, in a classic example of self-fulfilling methodology, the revascularization procedures that were done during the initial hospital stay did not count in the primary end point. Had these procedures been included in the analysis, the inference drawn from the study would have been completely different, since more than twice as many combined events of death, myocardial infarction, and revascularization would then be tallied in the group whose treatment was invasive as in the group whose treatment was ischemia-guided [43].

An additional argument marshaled in support of this approach is the open-artery hypothesis: the notion that beyond any consideration of myocardial salvage or symptomatic ischemia, there are long-term advantages to reopening an occluded or severely narrowed infarct-related coronary artery [44]. While this belief has been expressed for over a decade, there are as yet no prospective randomized trial data in its support. In fact, the only such study found paradoxically worse left ventricular remodeling in patients who underwent revascularization [45]. The ongoing NIH-sponsored Open Artery Trial will help clarify this question [46].

Thus, the previous restrained attitude that based invasive cardiac referral after MI on well-defined high-risk clinical criteria is being abandoned. Despite the paucity of data to support it and with little apparent concern for its economic and logistic implications, there are increasing calls to recognize systematic invasive cardiac management as the ideal option [47], one which has now become enshrined in the latest European Society of Cardiology guidelines on PCI as a class-1, level-of-evidence-A recommendation, with the dictum that if primary PCI cannot be performed, one should 'lyse now, stent later' [48].

### Unstable angina and non-ST-elevation myocardial infarction (NSTEMI)

#### Case 3

*A 49-year-old woman presented to a regional hospital with new, constant chest pain of several hours' duration. The ECG showed 1–2-mm inferior T-wave inversion. Treatment was initiated with nitrates, aspirin, and heparin. A few hours later, cardiac troponin became positive and a glycoprotein IIb/IIIa inhibitor was started as well as clopidogrel. Cardiac catheterization was requested at the nearest tertiary center, 100 miles away. The next day, with no recurrent symptoms, the patient was transferred. Angiography showed an occluded third marginal circumflex artery, stenosis of the distal left anterior descending artery estimated at 80%, and stenosis of the distal right coronary artery estimated at 70%. Angioplasty was performed, with insertion of two stents in the reopened marginal artery, three stents in the left anterior descending artery, and one stent in the distal right coronary artery. The subsequent radiological report evaluated the pre-PCI distal left anterior descending artery stenosis to be 40–50% and the pre-PCI distal right stenosis to be 50%*.

This case raises the questions of whether we are overtreating patients and overestimating their coronary lesion severity.

In parallel with the preeminence achieved by interventional cardiology in the management of STEMI, the pendulum has now swung in favor of the invasive strategy for patients with unstable angina and NSTEMI, much impelled by the recent FRISC-2 and TACTICS trials [49,50]. Earlier studies [51,52] suggesting that there is no advantage or even that there is a worse outcome with a systematically invasive strategy are now deemed irrelevant. The previous management strategy of reserving coronary angiography and revascularization for patients who were refractory to adequate medical therapy or were hemodynamically unstable or manifested evidence of low-threshold ischemia or signs of severe coronary artery disease on noninvasive testing is now considered too restrictive. What is the strength of evidence for this change?

The FRISC-2 study [50,53] randomized 2457 patients to either an invasive strategy with routine coronary angiography (and revascularization depending on the coronary artery anatomy) or initial noninvasive management. At one year, results favored the invasively treated group both in terms of death (2.2% versus 3.9%, *p *= 0.016) and death or recurrent MI (10.4% versus 14.1%, *p *= 0.005). A criticism of this study has been that its design disadvantaged the initially noninvasive (or 'conservative') arm because the clinical threshold for crossing over to the systematically invasive arm was too high. Patients had to have incapacitating symptoms or do very poorly on an exercise test (at least 3 mm ST-segment depression or limiting angina at low workload) to qualify for crossover. This is a higher threshold than most clinicians would normally tolerate and may have exposed these patients to avoidable risk. Indeed, this crossover threshold has been shown to miss about 50% of patients with severe coronary artery disease found by more conventional standards [54]. This finding underscores the limitations of the term 'conservative strategy', if the strategy is not anchored to clinically appropriate criteria for recourse to invasive cardiac procedures. Overly stringent criteria for crossover ensure a finding of superiority for a systematic invasive strategy, while overly unselective criteria lead to futile comparisons between strategies that end up resembling each other.

**Figure 1:**
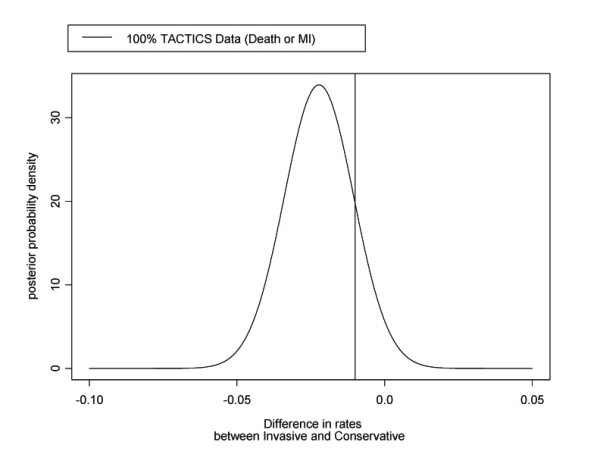
Probability density difference between invasive and conservative arms in TACTICS for combined death/MI outcome [49]. A binomial model is used for the control and intervention groups, and then, because the sample sizes were large, the posterior distribution, which is really the difference between two beta distributions, is approximated with a normal distribution. A Haldane prior (beta(0,0)), which contributes no information to the analysis, has been used. The area under the curve to the left of any given point represents the probability of the superiority of the invasive arm. For example, there is a 97% probability that the invasive arm is superior to the conservative arm (area under the curve to the left of the vertical line at 0). However, the probability that the benefit exceeds 1% or 2% is only 85% and 57%, respectively.

The TACTICS trial [49] also concluded that an early invasive strategy is superior, although the results found in this trial are quite modest relative to the impact it has had on the generalization of invasive cardiac management (Figure 1). The invasive strategy did not significantly reduce mortality, and the reduction in the combined end point of death or MI was of only borderline statistical significance at 6 months (7.3% in the invasive arm versus 9.5% in the conservative arm, *p *= 0.049). Limitations, including the previously described detection bias for MI [55] and unbalancing of adjunctive therapies following randomization (less platelet IIb/IIIa blockade during cross-over PCI in the conservative arm than in the invasive arm and more oral antiplatelet therapy in the invasive arm) cloud any interpretation of superiority. In support of these criticisms, the more recent RITA 3 trial [56] found no significant difference in the occurrence of death or MI with an invasive versus a conservative approach in patients with ACS at one year (RR 0.91; 95% CI 0.67–1.25). Only the softer end point of 'refractory angina' was reduced with an invasive strategy.

Notwithstanding the fragility of the above evidence in its favor, the recently updated ACC/AHA Guidelines now call strongly (Class I, Level of Evidence A) for an early invasive strategy in patients with unstable angina/NSTEMI who have any 'high-risk indicators' [57]. The need for early cardiac catheterization with likely revascularization is undisputed for refractory ischemia, arrhythmic or hemodynamic instability, ischemic heart failure, or evidence of severe disease on noninvasive testing. However, other postulated high-risk features such as PCI within 6 months or prior bypass surgery are intuitively questionable and suffer from a lack of evidence, because these patients have often been excluded from the major clinical trials. And while new ST-segment depression and/or raised cardiac troponin do indicate patients at higher risk, why is an initially conservative approach necessarily eschewed? Most such patients in fact do well, as suggested by the low positive predictive value (10–15%) of raised troponin for the end point of death or MI [58]. Consequently, the quasi-automatic recourse to invasive interventions and expensive drugs for all patients with ACS and raised troponin may be questioned. It is surprising that a major decision of clinical management now increasingly tends to be driven by the single reading of one blood test, without considering the patient's entire–and dynamic–clinical picture.

A very recent meta-analysis of 7 clinical trials (9212 patients) comparing routine and selective catheterization strategies in non-ST elevation ACS actually showed significantly increased mortality during hospitalization in the routine invasive arm and no significant overall mortality difference (odds ratio (OR) routine versus selective 0.92; 95% CI 0.77–1.09, p = 0.33) [59]. There was an absolute 2.1% fewer nonfatal MIs (OR 0.75; 95% CI 0.65–0.88, p < 0.001), and an absolute 2.8% less severe angina (OR 0.77; 95% CI 0.68–0.87, p < 0.001) with the routinely invasive strategy [59]. Statistical significance notwithstanding, these small clinical differences are further attenuated by the methodological limitations of TACTICS and FRISC-2 previously discussed. Therefore, we believe the evidence in favor of a systematic invasive strategy is not compelling.

This position is reinforced by two very recently published studies. The first is the long-term (3–5 years) follow-up of the RITA 3 trial [60]. It shows a rate of death or MI of 16.6% with systematically invasive treatment and 20.0% with conservative treatment (OR 0.78, 95% CI 0.61–0.99, *p *= 0.044). Although this would seem to support a blanket invasive approach, the small degree of benefit, an annualized rate difference of less than 1%, with a wide, barely statistically significant confidence interval, suggests that this evidence is not conclusive. Moreover, when the patients were stratified by baseline risk, only those in the eighth highest risk group appeared to benefit. This study can therefore be interpreted as an incentive precisely ***not ***to treat all patients with ACS invasively but rather to identify the minority that would truly benefit from invasive management. The second study, the ICTUS trial [61] of 1200 troponin-positive NSTEMI patients receiving the most contemporary medical and invasive management strategies, found no significant difference at one year in the combined end point of death, nonfatal MI, and rehospitalization for anginal symptoms between a systematic early invasive and a conservative strategy (1.07, 95% CI 0.87–1.33, *p *= 0.33). The mortality rate was the same and MI (applying the same definition to both groups) was actually more frequent in the group given systematically invasive treatment (15% versus 10%, *p *= 0.005). These findings were essentially unchanged when the definitions for the MI end point of the previous studies were applied.

### Cost-effective and real-world considerations

The socioeconomic aspects of the paradigm shift to systematically invasive management of ACS have been insufficiently addressed. PA is personnel-intensive, requiring multiple teams of skilled interventional cardiologists, nurses, radiology technicians, and physician assistants to provide round-the-clock service. It is also resource-intensive, requiring sophisticated nonreusable catheters, guide wires, stents, and adjunctive pharmacologic therapy. In the coming years, high-tech lassos, thrombotic aspirating devices, and drug-eluting stents will be increasingly added to this costly armamentarium. If patients are transferred for PA, additional resources of ambulances and qualified personnel will be required, often with return trips. Nor does this approach necessarily assure an uneventful follow-up. About 40% of these patients may be rehospitalized and about 20% may undergo a new revascularization procedure within the year [62,63]. Although the number of the latter will probably be reduced with the addition of drug-eluting stents, the rate of adverse events will likely still be about 20% [64]. Drug-eluting stents also increase the need for long-term multiple antiplatelet drugs to prevent late stent thrombosis.

Therefore, if the standard treatment of ACS is to be displaced from community hospitals to become obligatorily tertiary, the economic and logistic consequences on the health care system and society will be appreciable [65]. The absence of discussion of these issues is paradoxical when one considers the extensive debate that prevailed a decade ago over a far more modest cost consideration, namely, which of two very differently priced thrombolytic agents should be used. For example, does a possible small incremental benefit of PA, whose certainty may be doubted from the preceding discussion, compared with point-of-care thrombolysis in a patient with an uncomplicated acute MI justify the major additional resource costs?

As more patients undergo invasive management, the practice reinforces itself under the circular reasoning that, since most patients will be catheterized in any case, they should be catheterized as early as possible to derive benefit quickly, nothing being gained by delay or an expectant attitude. When comparative cost-effective analyses have been performed, they have tended counterintuitively to favor the invasive strategy, because the investigators have attributed the artificially high rates of intervention observed in their clinical trials (cardiac catheterization rates of 63–91% and revascularization rates of 49–73%) to the control group [49,66-69]. This is more because of clinical practice patterns than because of clinical need, as is attested by the marked variability of tertiary intervention noted in clinical studies (less than 10% in FRISC-2, several times more in TACTICS) [49,53], registries, and administrative databases [70]. However, studies comparing regional or national clinical practices have shown that higher rates of invasive procedures following MI, compared with much lower rates, do not improve survival, and affect reinfarction little or not all, to justify the considerable burden that generalized invasive management entails [71-74]. Importantly, other analyses suggest that the optimal rate of invasive intervention following MI in terms of outcome is 20–30%, beyond which there does not appear to be incremental benefit [75,76].

It is underappreciated that very recent and large registry studies actually lend support to a more selective rather than routine invasive approach in the management of acute coronary disease. GRACE, a prospective multinational observational registry study involving 28,825 patients with ACS, showed higher adjusted risks of death, stroke, and bleeding complications at 6 months in patients first admitted to hospitals with catheterization facilities than to hospitals without them, despite 10 times more revascularization procedures in the former hospitals (41% versus 3.9% for PCI and 7.1% versus 0.7% for coronary bypass surgery, respectively) [77]. This study calls into question the increasingly advocated notion that patients with ACS should be directed preferentially to tertiary centers for routine invasive treatment. In similar vein, a cohort study of 158,831 elderly Medicare patients hospitalized with acute MI and followed for 7 years found that routine use of more costly and invasive treatment strategies was not associated with a benefit beyond that seen with excellent medical management [78].

It is argued that expeditious invasive management of patients with ACS shortens hospital stays, rendering this approach cost-effective. According to this perspective, the problem is essentially the 'culprit lesion' and once it has been rapidly 'fixed', early discharge makes this approach superior to the more protracted scenario of medical stabilization, 'watchful waiting', and non-invasive stratification. However, algorithms shortening hospital stays with noninvasive approaches have been validated, although they receive less attention [79]. Studies that show reduced hospital stays with invasive management are necessarily unblinded, with possible introduction of bias.

### Philosophical and broader implications

The increased recourse to invasive management of acute coronary disease also raises provocative philosophical, intellectual, and social issues. First, there is a tendency to reduce acute coronary disease, and atherosclerosis in general, to the problem of a defective localized segment of a coronary conduit amenable to an expeditious mechanical fix. This perception is paradoxical given the growing understanding of the diffuse and inflammatory nature of the atherosclerotic disease process and the complex biology of the vascular wall, including recent data suggesting the simultaneous presence of multiple potential culprit plaques in acute coronary disease [80,81]. The current tendency to dilate coronary stenoses solely on the basis of their perceived severity (the so-called oculo-stenotic reflex) also disregards sound evidence that coronary lesions that are the substrate for ACS are quite often not severely stenosed, whereas some severe stenoses are known to remain stable for many years [82-85]. The reductionist perception also has far-reaching consequences for the quality of the patient-physician relationship, academic medicine, and patient and public comprehension of a very serious disease ('They unblocked my artery!'). Nor may it render a service to the patient in terms of adequately addressing the hardly negligible psychological dimension and the importance of organizing an effective and empowering strategy of prevention.

A second issue is the fundamental terminological trap inherent in the 'aggressive' versus 'conservative' debate on the management of acute coronary disease. When the medical problem is perceived as serious and high-risk, 'conservative' bears the stigmatism of being passé, conveying a sense of defeatism, grudging parsimony, doing less than the maximum best for the patient. If the 'conservative' approach fails, it confirms that the clinician was too passive and must assume the opprobrium, indeed may risk a lawsuit. 'Aggressive' implies courage and active will to engage an enemy disease, and enjoys the aura of power and high-tech glamor. If the intervention fails or provokes unfortunate side effects, blame is unlikely to accrue to the chooser of the aggressive course, vindicated because the problem was indeed serious, as events bore out, and all was done that could have been done. We must be aware how language and the unconscious cultural images it evokes condition our perceptions of patient management and the values we transmit to trainees, colleagues, patients, and the general public.

A third issue is the acceptance of a therapeutic attitude that justifies complex tertiary care intervention for a common medical condition for at most marginal clinical benefit. If it is granted–although we have argued this is debatable–that recourse to generalized invasive cardiac procedures leads to some meaningful health benefits, does it follow that we must accept the imposition of an enormous therapeutic burden on all because we are unable to use clinical judgment to identify more narrowly those most likely to benefit? Maseri has likened this problem to a hypothetical scenario where we would treat all patients who have anemia with iron or vitamin supplements [86]. While there would likely be a statistically significant benefit from such an intervention, this would be regarded as inappropriate clinical practice, because it disregards the notion that anemia may have many causes. Unselective, expensive treatments in acute coronary disease are not presently perceived as an imperfect approach and an implicit admission of clinical impotence [87].

Fourthly, generalized tertiary management of acute coronary disease has consequences for fragile health care systems and society. Where these resources are rarer, ready access for those especially in need of tertiary cardiac care risks being compromised because the queue has become too long and unselective. Necessary care for the less fortunate may be particularly jeopardized. The strain placed on already beleaguered universal health care systems may menace their integrity besides adversely affecting other health care domains and societal priorities [88].

Fifthly, interventional cardiology is highly remunerative in most medical systems based on fee for service, and tertiary cardiac centers clearly benefit from the contemporary approach to ACS. It has increasingly become the clinical practice that the diagnostic angiogram, the decision to do PCI, and the procedure itself are performed in the same session by the same individuals (judge, jury, and executioner?), creating a situation less propitious for considering other options such as simple abstention or recourse to cardiac surgery that have more clinical benefit in certain situations [89]. The direct and indirect conflicts of interest inherent in this situation and the manner in which this may affect how study findings have been interpreted and applied in clinical practice have generally been insufficiently acknowledged and ventilated. This problem is compounded by the increasing involvement of the cardiac-device industry in clinical studies and the multiple roles assumed by the same individuals as principal investigators, consultants, authors, opinion-leaders, and members of guidelines committees.

And finally, the invasively based homogenization of current medical practice in acute coronary disease and the pressures to conform that it has induced are abolishing the intellectual space that is normally occupied–and savored–by discriminating clinical judgment. Acute coronary care is at risk of becoming a far less attractive field to those seeking creative challenges as clinicians and students of pathophysiology. And if ACS becomes a tertiary disease, will recruitment of cardiovascular specialists to secondary hospitals not risk being jeopardized? Might this development not ultimately result in suboptimal treatment of cardiovascular disease in nontertiary settings [65] ? Moreover, questions about the continuity of care following the acute coronary episode have not been adequately addressed.

## Summary

In its relatively unselective plunge into technology, we believe contemporary cardiology has taken a turn that is problematic, on scientific, economic, and intellectual grounds. The capital notion that diagnostic procedures and treatments should be adapted to patients–and not patients to treatments–with their unique clinical particularities, differing underlying pathogenic mechanisms, and heterogeneous risk profiles, should not fall victim to a systematic steamroller approach in patients with ACS. We dare hope that the countervailing attitude expressed here may contribute to the salutary debate necessary for an equilibrium in which clinical finesse and discriminating judgment may assume their proper place in the management of patients with acute ischemic heart disease.

## Competing interests

PB received speaker fees at a few academic meetings sponsored by Hoffmann-La Roche Limited in 2004 and 2005. JMB has no competing interests.

## Authors' contributions

PB and JMB contributed equally to this work and have read and approved the final manuscript.

## Pre-publication history

The pre-publication history for this paper can be accessed here:
